# Open Surgery for Sportsman’s Hernia a Retrospective Study

**DOI:** 10.3389/fsurg.2022.893390

**Published:** 2022-06-16

**Authors:** Piergaspare Palumbo, Fanny Massimi, Sara Lucchese, Serena Grimaldi, Nicola Vernaccini, Roberto Cirocchi, Salvatore Sorrenti, Sofia Usai, Sergio Giuseppe Intini

**Affiliations:** ^1^ Department of Surgical Sciences, Sapienza University of Rome, Rome, Italy; ^2^ Department of General Surgery, University of Udine, Udine, Italy; ^3^ Department of Surgical and Biomedical Sciences, University of Perugia, Perugia, Italy

**Keywords:** sportsman hernia, groin pain syndrome, groin pain in athletes, open hernioplasty, laparoscopic hernia repair

## Abstract

Sportsman’s hernia is a painful syndrome in the inguinal area occurring in patients who play sports at an amatorial or professional level. Pain arises during sport, and sometimes persists after activity, representing an obstacle to sport resumption. A laparoscopic/endoscopic approach is proposed by many authors for treatment of the inguinal wall defect. Aim of this study is to assess the open technique in terms of safety and effectiveness, in order to obtain the benefit of an open treatment in an outpatient management. From October 2017 to July 2019, 34 patients underwent surgery for groin pain syndrome. All cases exhibited a bulging of the inguinal posterior wall. 14 patients were treated with Lichtenstein technique with transversalis fascia plication and placement of a polypropylene mesh fixed with fibrin glue. In 20 cases, a polypropylene mesh was placed in the preperitoneal space. The procedure was performed in day surgery facilities. Early or late postoperative complications did not occur in both groups. All patients returned to sport, in 32 cases with complete pain relief, whereas 2 patients experienced mild residual pain. The average value of return to sport was 34.11 ± 8.44 days. The average value of return to play was 53.82 ± 11.69 days. With regard to postoperative pain, no substantial differences between the two techniques were detected, and good results in terms of the resumption of sport were ensured in both groups. Surgical treatment for sportsman’s hernia should be considered only after the failure of conservative treatment. The open technique is safe and allows a rapid postoperative recovery.

## Introduction

Sportsman’s hernia is defined as a bulge at the posterior wall of the inguinal canal, sometimes associated with chronic pain in the inguinal and pelvic areas. The bulge is not a real hernia, but it is linked to a posterior wall weakness. Pain arises with a subtle, or less commonly, acute onset, and the symptoms include complaints by athletes who enjoy activities requiring rapid changes in direction and extreme overload, such as soccer, rugby, steeplechase, skiing, ice hockey, and cricket (1–3). Occasionally, they are associated with other pathologies characterized by inguinal and pelvic pain, adductor tendinopathy, osteitis pubis, and symphysis pubis diastasis (4).

Therefore, the sportsman’s hernia is considered within the definition of the groin pain syndrome (GPS). At present, a consensus for a more suitable terminology has not been achieved, and in a systematic review, Serner et al. (5) stressed the need to use a consistent terminology. In 2012, during the First Consensus Conference of the British Hernia Society in Manchester (6), the term Inguinal Disruption (ID) was adopted, describing the specific feeling reported by athletes, such as a sense of tension or increasing pain at the inguinal area occurring during or after intense sport, and without the presence of a real inguinal hernia. This definition however does not consider other potential causes of groin pain (GP). The multifactorial etiology, instead, includes several clinical features, and makes the diagnosis challenging for the surgeon. In the Agreement Meeting on Terminology and Definitions on Groin Pain in Athletes in Doha, Qatar (2014), Weir et al. attempted to order the existing definitions based on the use of a procedure such as the Delphi procedure (7). Their conclusions facilitated a classification system based on four categories, confirming the confusion in the terminology currently used.

In February 2016, the first Italian Consensus Conference (8) identified GPS as “each clinic features that patient complain at the level of the inguinal and pubic area, compromising the fulfilment of the sport activity and/or hinder the activities of daily living, and requiring medical attention”.

The true incidence of sportsman’s hernias is unknown, due to disagreements of terminology and diagnosis.

In most studies a prevalence of GPS in amateur athletes can be found; in fact, amateur athletes do not have the same access to suitable care, including conservative and surgical care (9, 10). By contrast, professional athletes are supported by a qualified medical staff.

Lovell (11) showed that sportsman’s hernias were first diagnosed in 95 (50%) of 189 cases of individuals complaining of chronic inguinal pain identifying a bulging of the posterior inguinal wall in 80% patients affected by unknown origin groin pain (12). In a prospective study carried out by Holmich (13) of 2017 patients with inguinal pain associated with sport, the mean age ranged from 26 to 28 years, and only 11 patients (5%) were females. A study carried out by Zoland et al. (14) on female patients, showed that nine women out of 17 had only a rectum muscle injury, while eight patients also had an inguinal, femoral, or obturator hernia.

The treatment for sportsman’s hernia is a complex question and requires different professional figures for a multidisciplinary approach. But not more than 40% of athletes treated only with conservative techniques resumed normal sports activities (1). Therefore, surgery is essential to repair the bulging od the posterior inguinal wall.

The aim of this study is the evaluation of a surgical anterior approach for the treatment of the sportsman’s hernia.

## Materials and Methods

A retrospective study was carried out by the Department of Surgical Sciences, Sapienza University of Rome, Italy, and the Department of General Surgery at the University of Udine, Italy. From October 2017 to July 2019, 53 patients with suspected clinical diagnosis of sportsman’s hernia were considered. The inclusion criteria were the professional or amateur level sport activity, age ranging between 18 and 70 years, a BMI <30, an American Society of Anesthesiologists (ASA) score I e II, the presence of long-standing groin pain syndrome (LSGPS) without other pathologies, and the presence of inguinal bulging at the clinical examination. Patients with other inguinal or pelvic diseases, such as pelvic or hip trauma (3 patients), overt inguinal or inguinoscrotal hernia (11 patients), and prior surgery in the inguinal region (3 patients) were excluded from our study and underwent different surgery or conservative treatment. Thirty-six cases were considered eligible to our analysis.

The diagnostic work up was carried out using dynamic ultrasonography of the abdominal wall, searching for the presence of a posterior wall bulging under Valsalva’s maneuver, confirming our clinical suspect, and detecting any possible contralateral defects. In the case of posterior inguinal wall bulging, patients underwent plan pelvic radiograph, “Flamingo stress view” and oblique radiograph of Dunn.

A pelvic MRI was performed as well. Two patients were excluded from surgery due to a pelvic imbalance and bone marrow edema. The main MRI findings were represented by rectus abdominis and adductor aponeurosis tears (31 patients, 91.2%), dehiscence of adductor tendons (8 patients, 23.5%), rectus abdominis edema (5 patients, 14.7%), pubic ligament insertion atrophy (2 patients, 5.8%). However, in all cases a bulging during Valsalva’s maneuver was present and identifiable during MRI.

A total of 34 patients were selected for surgery and sent to preoperative physiotherapy for eight weeks to focus on muscle stretching. No patients experienced lasting pain relief, and therefore, were sent to surgery. A comprehensive, informed consent was obtained. Patients were treated by open procedures with local anesthesia, and they were divided into two groups, based on the anterior or preperitoneal mesh placement; the surgical technique chosen depended on the team preferences and expertise. In 14 patients (41.2%) the chosen technique was a Lichtenstein hernioplasty with transversalis fascia plication and placement of a polypropylene light mesh fixed with fibrin glue (Tisseel®, Baxter Int., USA). In the other 20 cases (58.8%), a polypropylene light mesh was placed in the preperitoneal space, not using fixation devices. In both cases, therefore, no sutures were used. Both procedures were performed with an open access, as outpatient, with the aim of discharging the patients 4 h after surgery. A modified Post Anesthesia Discharge Scoring System (PADSS) (15) was administered before patient discharge to ensure their safe return home. Furthermore, all patients were informed about the recovery time and caution required to achieve a postoperative course free of complications and a quick resumption of sport.

Patients were requested to undergo physiotherapy sessions beginning 2 weeks after surgery to strengthen the hips and thigh muscles, and in particular, the adductors, quadriceps, rotators, and iliopsoas muscle. At the same time, patients were invited to carefully start again sports activities. Sudden movements were prohibited; the only sport allowed was the brisk walk, progressing to jogging and running over 3–4 weeks. Special activities, such as running on a track and performing sprints could be resumed from the twentieth postoperative day onward.

After discharge, all patients were followed up at 48 h, at one week, and after one month, to rule out the presence of early complications. The postoperative pain was assessed with a 0–10 Numerical Rating Scale test (NRS) administered at 48 h and a week after the surgical procedure. Moreover, all patients underwent a postoperative rehabilitation program from the next 48 h after surgery.

The rehabilitation program is divided into progressive modules and tailored depending on the needs of the patient. It lasted about 3 months after surgery and was aimed to reduce pre-existing risk factors and whenever not possible, to learn compensatory strategies, improve the core stabilization and strength around the pelvis particularly in single-leg stance.

In our rehabilitation program core exercises represented the first step, starting with static activation of the core muscles and gradually progressing by adding limb movements in stable positions and more unstable positions, until reaching more functional and dynamic positions.

Physiotherapists paid particular attention to the development of a strong single-leg position and to the adductor strengthening, progressively adding external load.

Moreover, muscle stretching was routinely performed.

The physiotherapists were also present in the first phases of the return to sport, to directly supervise all the progress and any issues or change in the personal rehabilitation program. Particularly, each patient was asked to report any adverse response within 3 days of rest after the training in order to tailor its program, sometimes returning to the previous level of activity. To progress in their sports, the training should be pain-free.

The Return to Sport (RTS), indicating the time to onset of training in their respective sports activities, and Return to Play (RTP), meant as the time to acquire the ability to play obtaining the best results, were evaluated.

The total follow up was extended for three months after surgery.

The statistical analysis has been conducted using the Student t-test. The Normality test (Shapiro-Wilk test), has been conducted due to the small sample size, with a *p*-value = 0.451 and W = 0.877607

## Results

Thirty-six patients were included in the study, whose main characteristics are presented in Table 1. All patients were male, with a median age 29 years, and a median BMI of 22 kg/m^2^. Thirty patients played football, four played football and dance, one played football and judo, and one boxing.

**Table 1:** Main characteristics of the population object of study.

Characteristics of patients (*n*)	*n* = 36
Sex (M, F, %)	M, 100
Age (mean, years)	28.55 ± 8.67
BMI (mean, kg/m^2^)	23.68 ± 2.49
Sport activity (*n*)	
Football	30
Football + dance	4
Football + Judo	1
Boxing	1
Professional (%)	22.2
Duration of symptoms (mean, weeks)	12.71 ± 9.14
Pain localization at clinical examination (%)	
Insertion of the rectum on the pelvis	79.4
Superficial inguinal ring	55.8
Sports activities withdrawal (%)	47.3
Hindered daily activities (%)	2.8

These symptoms and diagnosis are unusual in a boxer, but in this case, he used to train also by racing, so that it’s more likely that the principal aethiology of the groin pain syndrome was not the sport practiced but rather the way he trained.

All patients complained of pelvic pain that increased during physical stress. Symptoms lasted a median of 14 weeks and in the meantime, 52.7% of the patients continued to play their respective sports. Only in one case (2.8%) daily activities were partially hindered.

Pain occurred mainly at the insertion of the rectum on the pelvis (79.4%) and/or at the superficial ring (55.8%), particularly while under strain or when coughing. None of the patients had relevant comorbidities.

Dynamic ultrasonography showed a bulge of the posterior inguinal wall in 100% of cases. In one case, a retraction of the long adductor insertion was revealed. After the imaging study, 34 patients were considered eligible to surgical treatment. In the group treated with anterior inguinal hernioplasty, one patient underwent a partial tenotomy of the proximal insertion of the long adductor. None of the patients underwent bilateral hernia repair. The mean time of the surgical interventions was 56.42 ± 8.32 min for the anterior approach and 44.5 ± 12.5 min for the posterior one (*p* = 0.79). All patients were discharged 4 h after surgery, without immediate complications. The PADSS was administered before discharge to ensure their safe return home. Postoperative pain was treated with 1 g of Paracetamol, two times per day.

None of the patients presented severe postoperative complications such as hemorrhage requiring a surgical second-look, deep venous thrombosis and/or pulmonary embolism, bowel or bladder injury or ischemic orchitis. Early complications, defined as complications arising up to 30 days after surgery, occurred in two patients treated by anterior approach (14.29%), in which a superficial seroma was observed without requiring treatment.

The NRS values were comparable in both techniques. The average value of NRS at 48 h was 2.71 for the anterior approach, and 2.50 for the posterior one (*p* = 0.99). The average value of NRS a week later was 1.71 for the anterior technique, and 1.35 for the posterior one (*p* = 0.58)

A total of 91.17% (31 patients) of patients followed the rehabilitative protocol and all patients returned to their respective sport. In two cases (amatorial sportsmen), the final outcome was satisfactory in terms of resumption of sport, but the remission of pain was incomplete.

The median value of RTS was 35 days. The RTS following the anterior approach was 34.28 ± 5.62 days, while that following the posterior approach was 34.0 ± 9.94 days (*p* = 0.92).

**Figure 1:**
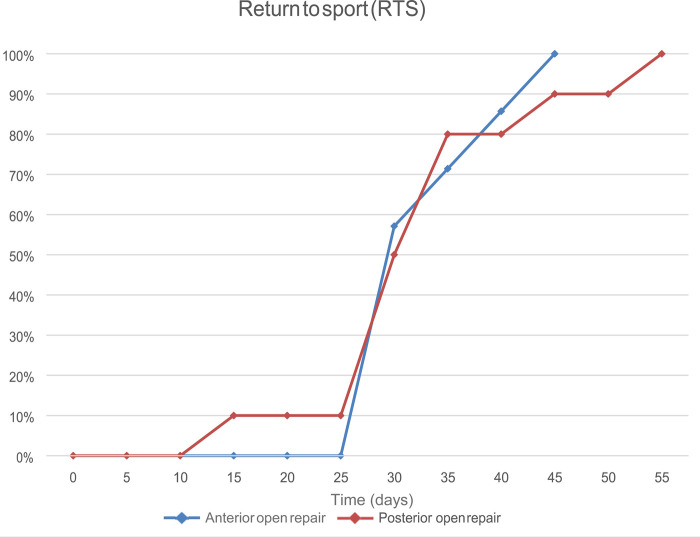
Return to Sport in terms of days in both groups.

**Figure 2:**
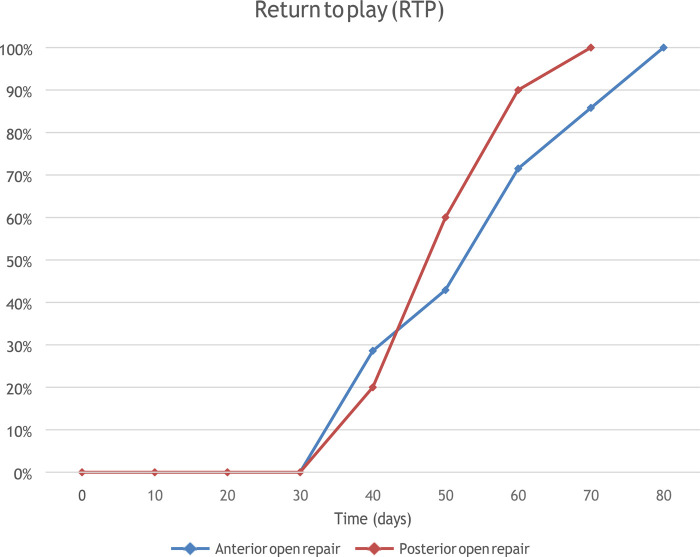
Return to Play in terms of days in both groups.

The median value of RTP was 56 days. The average RTP after anterior treatment was 57.14 ± 13.85 days, while after the posterior approach it was 51.50 ± 9.23 days (*p* = 0.16). Results are reported in Table 2 and Figures 1, 2.

**Table 2:** All intra-operative and post-operative items evaluated in both groups.

Surgical technique (*n* = 34 patients)	Anterior approach (14 patients, 41.2%)	Posterior approach (20 patients, 58.8%)	*p*-value
Operating time (mean, min)	56.42 ± 8.32	44.5 ± 12.5	0.79
Postoperative pain, NRS at 48 h (mean)	2.71	2.50	0.99
Postoperative pain, NRS at 1 week (mean)	1.71	1.35	0.58
Early complications, *n* (%)	2 (14.29)	0 (0)	0.32
Late complications (%)	0	0	0.99
Chronic neuralgia (%)	0	0	0.99
RTS (mean, days)	34.28 ± 5.62	34.0 ± 9.94	0.92
RTP (mean, days)	57.14 ± 13.85	51.50 ± 9.23	0.16

*NRS, numerical rating scale; RTS, return to sport; RTP, return to play.*

The follow-up evaluations until present did not reveal any cases of late complications and chronic neuralgia, for both open surgical techniques.

## Discussion

The cause of sportsman’s hernia is unknown, and the multiple existing theories seem to explain only part of GPS (2–4).

To get an effective diagnosis, it is necessary to exclude all other conditions of the joints (acetabular injuries, femoro-acetabular impingement (FAI), Legg-Calve-Perthes Disease), visceral causes (inguinal and abdominal hernias, bowel diseases), bones (fractures, pubic osteitis, pubic symphysis diastasis), muscle-tendons (rectum and adductor tendons injuries, iliopsoas injuries, bursitis), neurological causes (nervous entrapment syndrome), genitourinary diseases (varicocele, testicular/ovarian torsion, pelvic inflammatory disease, ovarian cysts, prostatitis, orchitis and epididymitis, and cystitis), cancers, infections, rheumatic diseases, and lymphatic causes (8).

Symptoms can be bilateral in up to 12% of cases. Pain involves, typically, the pubic area with irradiation to the adductor region in 40% of cases and to the inguinal, and the perineal and genital area in 6% of cases only, with an insidiously onset in 2/3 of patients (16). Consequently, a surgical repair procedure for the bilateral bulging (if present) should be considered only in such cases. In a study published by Meyers et al. (17), 60% of patients initially complaining of inguinal pain, also later showed a painful symptomatology localized only at the proximal insertion of the adductor muscle.

The symptoms at onset of sportsman’s hernia are variable and can arise during warmup and attenuate during gameplay or can occur only during gameplay and regress with rest (18). Pain is often worsened by sudden accelerations, rotation, kicking movements, sit- ups, coughing, or sneezing (19). Among the footballers affected by GPS, 32% feel pain when kicking (20). Diagnosis is based on precise anamnesis and a rigorous examination at rest, with tests focused on muscle contractions, active and passive stretching procedures (21), and palpation targeted to specific anatomical areas (22, 23). Sportsman’s hernia should be considered in the presence of unexplainable chronic inguinal pain, with tenderness to palpation of the inguinal region, and possible irritation to the inner thigh region (24, 25). A sit-up against resistance (crunch test) during palpation of the inferolateral margin of the distal rectum muscle, or the Valsalva maneuver can reduce symptoms (25).

Diagnostic imaging of GPS is of fundamental importance. According to the first Italian Consensus Conference (8), the diagnostic images must be based on conventional radiology, ultrasound, and MRI. Radiological imaging includes X-rays of the pelvis in an anteroposterior projection to show possible bony erosions or a dysmetria of the pubic branches, acetabular retroversion (PINCER-FAI), osteoarthritis (it could be evident also in young people), tumors, and stress fractures. The projection in alternate support (“Flamingo views”) is also needed to search for pubic symphysis instability, and the Dunn’s oblique projection is used for the assessment of a possible femoro-acetabular impingement (FAI deformity *α* angle >55°).

Basal and dynamic ultrasound allows for the assessment of abdominal and adductor muscle tendons, and the evaluation of the inguinal canal posterior wall and structures, the urinary tract, and external genitalia.

MRI can provide detailed information concerning bones, tendons, and muscles and is useful to identify the presence of bone marrow edemas (BME) at the pubic branch.

The sportsman’s hernia treatment requires a multidisciplinary approach and all therapies supporting surgical procedures, including active or passive physiotherapy, shock waves, and injections of platelet enriched plasma (PEP) performed before and after surgery are essential for the optimal functional recovery of the patient.

Such treatments must be tailored on the patients’ clinical history, its sport level and on the results of diagnostic tests and should be aimed at the functional recovery and rapid RTS for the patient, with the purpose to minimize the absence of the athlete from sport, as well as improving performances (26, 27).

Physiotherapy and rehabilitation with exercises to reinforce the abdominal wall, lumbar region, and coxo-femoral joint are recommended.

A systematic review published by Jørgensen et al. (24) in 2019 showed that conservative treatments (physiotherapy, steroid, and analgesic injections, or watchful waiting) should have a duration of 2–6 months. Conversely, other studies established the superiority of surgery over conservative treatments (5, 24, 28, 29).

In a study conducted by Paajanen et al. (28) comparing surgery to conservative treatment, the GP reduction at 1 and 12 months after surgery was more significant than the GP reduction of patients in the conservative-treatment group (*p* < 0.001) and 90% of patients returned to sport after three months from surgery compared 27% of the athletes in the conservative-treatment group (*p* < 0.0001).

Ekstrand et al. (29) compared the outcomes of patients submitted to surgery with those who underwent physiotherapy or watchful waiting, showing that the surgery group experienced significant pain reduction compared to the other groups. However, they recommend surgery only after conservative treatments failure (with pain persistence after physical therapy for 2 months) and after at least 3 months from the onset of symptoms.

According to several authors, surgery should be reserved only in case of evident bulging, enforcing the transversalis fascia, similar to conventional groin hernioplasty (4, 15, 30), and the choice of open or laparoscopic approach (transabdominal pre-peritoneal, TAPP, or totally extra-peritoneal, TEPP hernia repair) is at the discretion of the surgeon. Considering the return to sport as the only outcome, surgery seems to be most effective, regardless of whether it is open or laparoscopic (4).

Open hernioplasty without the use of prosthetic devices was proposed by Litwin et al. (27), and patients exhibited improvements in physical scores (15.5%), and mental scores (7.8%), while a small percentage of patients were subsequently subjected to a second look and/or hip arthroscopy.

The Minimal Repair Technique (MRT) does not require the use of mesh as well as the Shouldice one, and it is based on the assumption that GPS is caused by a limited weakness of the posterior wall of the inguinal canal, resulting in the entrapment of the genital branch of the genitofemoral nerve. However, the suture may determine a power imbalance on the pubic symphysis (rectus abdominis versus adductor) and leads to a chronic inflammatory condition that acts directly on the sensitive branches of the nerves (31, 32, 33). Hernioplasty using prosthetic devices is the most common surgical procedure, ensuring a tension-free repair process and reducing the complications associated with the suture (i.e., the recurrence of postoperative chronic pain) (34, 35). The hernioplasty can be associated with adductor release in case of GPS associated to tendinopathy, aiming to reduce the functional imbalance between abdominal and adductor muscles (4, 18). Although a tendinopathy of the long adductor muscle can be diagnosed in 40% of patients, only 18% of those individuals need a tenotomy. Keeping the muscle belly intact and minimizing the loss of strength, long adductor tenotomy is realized through a series of small incisions over the epimysium for about 3 cm (36).

In this study, the results after the open anterior and posterior surgical techniques were compared, analyzing how a different approach to the inguinal nerves can lead to a possible appearance of postoperative pain or chronic neuralgia, invalidating the results of surgery. A careful identification of the nerves allowed to limit any damage in both cases, although the mesh placed back to the transversalis fascia potentially should avoid direct contact with the nerves (37). However, no differences between the two techniques were detected, and good results in terms of the RTS and RTP (in line with the reported literature) were obtained, allowing a fast recovery. The use of loco-regional anesthesia and a less invasive surgical technique (both anterior and posterior) allowed a rapid discharge of all patients and a quick return to their daily routine without compromising or delaying the resumption of the postoperative physical rehabilitation. Furthermore, in terms of early and late postoperative complications, all the results obtained showed no statistically significant differences between the two groups, showing that both techniques are safe and effective, and no patients reported at the postoperative 3-months follow up persistent groin pain or neuralgia.

The defect on the abdominal wall can be also treated using a laparoscopic approach, with TAPP (19) o endoscopic with TEP (38), associating or not the Lloyd Release procedure (39). The release of the inguinal ligament at the level of the pubic tubercle could determine an improvement in the symptoms in such patients (40). More than 90% of whom were able to return to sport within an average of 4 weeks. These techniques are more invasive and require a specific learning curve.

Currently, based on data in the literature, there is not an approach for repairing sportsman’s hernia that is better than another. The studies and reviews available rarely report comparisons between techniques, suggesting that GPS treatment—whether open or laparoscopic—is often based on the surgical team experience. Thus, additional studies are required to compare the main surgical approaches and their outcomes.

According to Caudill et al. (4) the results achieved using open and laparoscopic techniques, in terms of a patients’ return to sport, were similar. Other authors reported a faster return to full sport following laparoscopic surgery, which ranged between 4 and 6 weeks (41). Neumayer at al (42). revealed a higher rate of GPS recurrence (10.1%) following laparoscopic surgery compared to open prosthetic hernioplasty (4.9%). Recently, Sheen et al. (43) published a European multi-center randomized study comparing the minimal repair technique and TEP, whose results showed a reduction of preoperative pain 4 weeks after surgery in both groups (*p* < 0.001). Moreover, the TEP approach proved to be less painful than the minimal repair technique during the first month, whereas both procedures are similarly effective in treating chronic pain.

Jørgensen et al. (24) compared different types of treatments (hernioplasty, adductor tenotomy, combined surgery and conservative treatment) and showed that the combined surgery (hernioplasty and long adductor release) was the most effective. According to Ahumada et al. (44), the open technique led to excellent results (83.3% of patients), but almost complete rest was required during the first 4 weeks after surgery;on the contrary, Ingoldby et al. (45) reported a return to sport within 4 weeks in 92.9% of athletes that underwent laparoscopic repair showing a faster recovery after a laparoscopic approach. No evidence is available on the necessity to repair an asymptomatic contralateral posterior wall defect. In a European survey, only 5% of surgeons were found to repair a contralateral defect (2). Nam et al. (46) reported that no more than 10% of athletes complained of pain within 10 years in the groin region which didn’t undergo surgery.

Postoperative physical rehabilitation is fundamental to the recovery process. Patients are recommended to begin walking immediately after surgery and progressing from jogging to running within 3–4 weeks. Running on straight tracks and sprints should be performed after the 21^st^ postoperative day and athletes usually return to full activity within 2–4 months after operation (4). Hemingway et al. (47) described a 6-week physical rehabilitation program following open surgery.

To date, there is no consensus on the best approach between open or laparoscopic surgery in the repair of sportsman’s hernia, as both procedures have strengths and weaknesses. The laparoscopic technique requires general anesthesia and is a more complex surgical procedure which requires a long learning curve, but the ileopubic tract, pectineal fascia, and origin of the rectus abdominis can be easily assessed. Furthermore, this approach allows the evaluation and repair of the contralateral inguinal region, although this condition is uncommon and does not affect pain persistence. On the other side, both the open techniques described, performed under local anesthesia, are minimally invasive, need a short hospital stay (2–4 h), and allow the surgeon to view both the rectus abdominis and conjoint tendon insertion on the pubic tubercle, so that a long adductor tenotomy might be performed, when appropriate. No differences have been detected comparing the anterior and the posterior open approach, mainly in terms of postoperative pain and persistent neuralgia.

## Data Availability

The raw data supporting the conclusions of this article will be made available by the authors, without undue reservation.

## References

[1] Paksoy M Sekmen U. Sportsman hernia; the review of current diagnosis and treatment modalities. Turkish J Surg. (2015) 32:122–9. 10.5152/UCD.2015.3132 PMC4942157 27436937

[2] Kingston JA Jegatheeswaran S Macutkiewicz C Campanelli G Lloyd DM Sheen AJ. A European survey on the aetiology, investigation and management of the “Sportsman’s Groin”. Hernia. (2014) 18:803–10. 10.1007/s10029-013-1178-4 24249070

[3] Rossidis G Perry A Abbas H Motamarry I Lux T Farmer K Laparoscopic hernia repair with adductor tenotomy for athletic pubalgia: an established procedure for an obscure entity. Surg Endosc. (2015) 29:381–6. 10.1007/s00464-014-3679-3 24986020

[4] Caudill P Nyland J Smith C Yerasimides J Lach J. Sports hernias: a systematic literature review. Br J Sports Med. (2008) 42:954–64. 10.1136/bjsm.2008.047373 18603584

[5] Serner A Van Eijck CH Beumer BR Hölmich P Weir A DeVos RJ. Study quality on groin injury management remains low: a systematic review on treatment of groin pain in athletes. Br J Sports Med. (2015) 49:813. 10.1136/bjsports-2014-094256 25633830 PMC4484372

[6] Sheen AJ Stephenson BM Lloyd DM Robinson P Fevre D Paajanen H “Treatment of the Sportsman’s groin”: British Hernia Society’s 2014 position statement based on the Manchester Consensus Conference. Br J Sports Med. (2014) 48:1079–87. 10.1136/bjsports-2013-092872 24149096

[7] Weir A Brukner P Delahunt E Ekstrand J Griffin D Khan KM Doha agreement meeting on terminology and definitions in groin pain in athletes. Br J Sports Med. (2015) 49:768–74. 10.1136/bjsports-2015-094869 26031643 PMC4484366

[8] Bisciotti GN Auci A Bona S Bisciotti A Bisciotti A Cassaghi G Groin Pain Syndrome Italian Consensus Conference on terminology, clinical evaluation and imaging assessment in groin pain in athlete. BMJ Open Sport Exerc Med. (2016) 2(1):e000142. 10.1136/bmjsem-2016-000142 28890800 PMC5566259

[9] Sheen AJ Iqbal Z. Contemporary management of “Inguinal disruption” in the sportsman’s groin. BMC Sports Sci Me Rehabil. (2014) 6:4–7. 10.1186/2052-1847-6-4 PMC4417524 25937929

[10] Diesen DL Pappas TN. Sports Hernias. Adv Surg. (2007) 41:177–87. 10.1016/j.yasu.2007.05.011 17972564

[11] Lovell G. The diagnosis of chronic groin pain in athletes: a review of 189 cases. Aust J Sci Med Sport. (1995) 27:76–9. PMID: 8599748

[12] Malycha P Lovell G. Inguinal surgery in athletes with chronic groin pain: the ‘sportsman’s’ hernia. Aus. N Z J Surg. (1992) 62:123–5. 10.1111/j.1445-2197.1992.tb00009.x 1586300

[13] Hölmich P. Long-standing groin pain in sportspeople falls into three primary patterns, a “clinical entity” approach: a prospective study of 207 patients. Br Sports Med. (2007) 41:247–52. 10.1136/bjsm.2006.033373 PMC2658954 17261557

[14] Zoland MP Iraci JC Bharam S Waldman LE Koulotouros JP Klein D. Sports hernia/athletic pubalgia among women. Orthop J Sport Med. (2018) 6:1–8. 10.1177/2325967118796494 PMC6144524 30246043

[15] Palumbo P Tellan G Perotti B Pacilè MA Vietri F Illuminati G. Modified PADSS (Post Anaesthetic Discharge Scoring System) for monitoring outpatient discharge. An It Chir. (2013) 84:661–5. PMID: 23165318

[16] Gilmore J. Groin pain in the soccer athlete: fact, fiction, and treatment. Clin Sports Med. (1998) 17:787–93. 10.1016/S0278-5919(05)70119-8 9922902

[17] Meyers WC Foley DP Garrett WE Lohnes JH Mandlebaum BR. Management of severe lower abdominal or inguinal pain in high-performance athletes. Am J Sports Med. (2000) 28:2–8. 10.1177/03635465000280011501 10653536

[18] Garvey JFW Read JW Turner A. Sportsman hernia: what can we do? Hernia. (2010) 14:17–25. 10.1007/s10029-009-0611-1 20066552

[19] Elattar O Choi HR Dills VD Busconi B. Groin injuries (Athletic Pubalgia) and return to play. Sports Health. (2016) 8:313–23. 10.1177/1941738116653711 27302153 PMC4922526

[20] Evans DS. Laparoscopic transabdominal pre-peritoneal (TAPP) repair of groin hernia: one surgeon’s experience of a developing technique. Ann R Coll Surg Engl. (2002) 84:393–8. 10.1308/003588402760978184 12484578 PMC2504190

[21] Campanelli G. Pubic inguinal pain syndrome: the so-called sports hernia. Hernia. (2010) 14:1–4. 10.1007/s10029-009-0610-2 20052510

[22] Preskitt JT. Sports Hernia: the experience of Baylor University Medical Center at Dallas. Baylor Univ Med Cent Proc. (2011) 24:89–91. 10.1080/08998280.2011.11928689 PMC3069511 21566750

[23] Gilmore CJ Diduch DR Handley MV Hanks JB. Sports Hernia and Athletic Pubalgia: Diagnosis and Treatment. In: Diduch D Brunt L, editors. Sports hernia-history and physical examination: making the diagnosis with confidence. Boston, MA: Springer (2014). p. 75–85.

[24] Jørgensen SG Öberg S Rosenberg J. Treatment of longstanding groin pain: a systematic review. Hernia. (2019) 23:1035–44. 10.1007/s10029-019-01919-7 30820781

[25] Larson CM. Sports hernia/athletic pubalgia: evaluation and management. Sports Health. (2014) 6:139–44. 10.1177/1941738114523557 24587864 PMC3931344

[26] Campbell KJ Boykin RE Wijdicks CA Erik Giphart J LaPrade RF Philippon MJ. Treatment of a hip capsular injury in a professional soccer player with platelet-rich plasma and bone marrow aspirate concentrate therapy. Knee Sur. Sports Traumatol Arthrosc. (2013) 21:1684–8. 10.1007/s00167-012-2232-y 23052123

[27] Litwin DEM Sneider EB McEnaney PM Busconi BD. Athletic Pubalgia (Sports Hernia). Clin Sports Med. (2011) 30:417–34. 10.1016/j.csm.2010.12.010 21419964

[28] Paajanen H Brinck T Hermunen H Airo I. Laparoscopic surgery for chronic groin pain in athletes is more effective than nonoperative treatment: a randomized clinical trial with magnetic resonance imaging of 60 patients with sportsman’s hernia (athletic pubalgia). Surgery. (2011) 150:99–107. 10.1016/j.surg.2011.02.016 21549403

[29] Ekstrand J Ringborg S. Surgery versus conservative treatment in soccer players with chronic groin pain: a prospective randomised study in soccer players. Eur J Sport Traumatol Relat Res. (2001) 23:141–5.

[30] Hopkins JN Brown W Lee CA. Sports hernia: definition, evaluation, and treatment. JBJS Rev. (2017) 5:6. 10.2106/JBJS.RVW.17.00022 28937419

[31] Comin J Obaid H Lammers G Moore J Wotherspoon M Connell D. Radiofrequency denervation of the inguinal ligament for the treatment of ‘Sportsman's Hernia’: a pilot study. Br Sports Med. (2013) 47:380–6. 10.1136/bjsports-2012-091129 22952407

[32] Muschaweck U Berger L. Minimal Repair technique of sportsmen’s groin: an innovative open-suture repair to treat chronic inguinal pain. Hernia. (2010) 14:27–33. 10.1007/s10029-009-0614-y 20063110

[33] Muschaweck U Berger LM. Sportsmen’s groin-diagnostic approach and treatment with the minimal repair technique: a single-center uncontrolled clinical review. Sports Health. (2010) 2:216–21. 10.1177/1941738110367623 23015941 PMC3445105

[34] Treadwell J Tipton K Oyesanmi O Sun F Schoelles K. Surgical options for inguinal hernia: comparative effectiveness review. Agency Healthc Res Qual Comp. (2012) 70(1):1219. PMID: ; Bookshelf ID: NBK100633 22993867

[35] Brown RA Mascia A Kinnear DG Lacroix V Feldman L Mulder DS. An 18-year review of sports groin injuries in the elite hockey player: clinical presentation, new diagnostic imaging, treatment, and results. Clin J Sport Med. (2008) 18:221–6. 10.1097/JSM.0b013e318172831a 18469562

[36] Brunt M Barile R. My approach to athletic pubalgia. In: Byrd J, editor. Operative hip arthroscopy. Springer (2013). p. 55–65.

[37] Cirocchi R Henry BM Mercurio I Tomaszewski KA Palumbo P Stabile A Is it possible to identify the inguinal nerves during hernioplasty? A systematic review of the literature and meta-analysis of cadaveric and surgical studies. Hernia. (2019) 23:569–81. 10.1007/s10029-018-1857-2 30570686 PMC6586705

[38] Voorbrood CEH Goedhart E Verleisdonk EJMM Sanders F Naafs D Burgmans JPJ. Endoscopic totally extraperitoneal (TEP) hernia repair for inguinal disruption (Sportsman’s hernia): rationale and design of a prospective observational cohort study (TEP-ID-study). BMJ Open. (2016) 1(6):e010014. 10.1136/bmjopen-2015-010014 PMC4716196 26739740

[39] Rennie W Lloyd D. Sportsmans groin: the inguinal ligament and the lloyd technique. J Belgian Soc Radiol. (2018) 101:2–7. 10.5334/jbr-btr.1404 PMC6251070 30498808

[40] Lloyd DM Sutton CD Altafa A Fareed K Bloxham L Spencer L Laparoscopic inguinal ligament tenotomy and mesh reinforcement of the anterior abdominal wall: a new approach for the management of chronic groin pain. Surg Laparosc Endosc Percutaneous Tech. (2008) 18:363–8. 10.1097/SLE.0b013e3181761fcc 18716535

[41] Piozzi GN Cirelli R Salati I. Laparoscopic approach to inguinal disruption in athletes: a retrospective 13-year analysis of 198 patients in a single-surgeon setting. Sports Med Open. (2019) 5:25. 10.1186/s40798-019-0201-4 31236737 PMC6591337

[42] Neumayer L Giobbie-Hurder A Jonasson O. Open mesh versus laparoscopic mesh repair of inguinal hernia. N Engl Med. (2004) 350:1819–27. 10.1056/NEJMoa040093 15107485

[43] Sheen AJ Montgomery A Simon T Ilves I Paajanen H. Randomized clinical trial of open suture repair versus totally extraperitoneal repair for treatment of sportsman’s hernia. Br J Surg. (2019) 106:837–44. 10.1002/bjs.11226 31162653

[44] Ahumada LA Ashruf S Espinosa-De-Los-Monteros A. Athletic pubalgia: definition and surgical treatment. Ann Plast Surg. (2005) 55:393–6. 10.1097/01.sap.0000181344.22386.fa 16186706

[45] Ingoldby CJ. Laparoscopic and conventional repair of groin disruption in sportsmen. Br J Surg. (1997) 84:213–5. 10.1046/j.1365-2168.1997.02460.x 9052437

[46] Nam A Brody F. Management and therapy for Sports Hernia. J Am Coll Surg. (2008) 206:154–64. 10.1016/j.jamcollsurg.2007.07.037 18155582

[47] Hemingway AE Herrington L Blower AL. Changes in muscle strength and pain in response to surgical repair of posterior abdominal wall disruption followed by rehabilitation. Br J Sports Med. (2003) 37:54–8. 10.1136/bjsm.37.1.54 12547744 PMC1724590

